# Fatigue Induced Changes in Muscle Strength and Gait Following Two Different Intensity, Energy Expenditure Matched Runs

**DOI:** 10.3389/fbioe.2020.00360

**Published:** 2020-04-22

**Authors:** Sherveen Riazati, Nick Caplan, Marcos Matabuena, Philip R. Hayes

**Affiliations:** ^1^Department of Sport and Exercise Sciences, School of Life Sciences, Northumbria University, Newcastle upon Tyne, United Kingdom; ^2^CiTIUS (Centro Singular de Investigación en Tecnoloxías Intelixentes), University of Santiago of Compostela, Santiago de Compostela, Spain

**Keywords:** running, running injury, running mechanics, HIIT, kinematics, running variability, force production, hip

## Abstract

**Purpose:**

To investigate changes in hip and knee strength, kinematics, and running variability following two energy expenditure matched training runs; a medium intensity continuous run (MICR) and a high intensity interval training session (HIIT).

**Methods:**

Twenty (10 Females, 10 Males) healthy master class runners were recruited. Each participant completed the HIIT consisting of six repetitions of 800 m with a 1:1 work: rest ratio. The MICR duration was set to match energy expenditure of the HIIT session. Hip and knee muscular strength were examined pre and post both HIIT and MICR. Kinematics and running variability for hip and knee, along with spatiotemporal parameters were assessed at start and end of each run-type. Changes in variables were examined using both 2 × 2 ANOVAs with repeated measures and on an individual level when the change in a variable exceeded the minimum detectable change (MDC).

**Results:**

All strength measures exhibited significant reductions at the hip and knee (*P* < 0.05) with time for both run-types; 12% following HIIT, 10.6% post MICR. Hip frontal plane kinematics increased post run for both maximum angle (*P* < 0.001) and range of motion (*P* = 0.003). Runners exhibited increased running variability for nearly all variables, with the HIIT having a greater effect. Individual assessment revealed that not all runners were effected post run and that following HIIT more runners had reduced muscular strength, altered kinematics and increased running variability.

**Conclusion:**

Runners exhibited fatigue induced changes following typical training runs, which could potentially present risk of injury development. Group and individual assessment revealed different findings where the use of MDC is recommended over that of *P*-values.

## Introduction

Running has been identified as one of the top five most popular leisure time physical activities worldwide (Hulteen et al., 2017). The growing popularity of club recreational running has, in part, been due to community organised, small weekly 5 km running event such as the Parkrun (Wiltshire et al., 2017). There has however been a concurrent increase in the incidence of running related injuries (RRI) with Linton and Valentin (2018) reporting that approximately 50% of Parkrun participants experience a type of RRI and continue running despite of the injury. Injuries can have a significant negative economic consequence through mental well-being, direct health care costs and loss of paid work (Hespanhol Junior et al., 2016). Epidemiological studies have reported a RRI incidence up to 70% (Buist et al., 2010) and up to 79.3% of the injuries occur in the lower extremities, with the knee (up to 50%) the most common site of injury (Van Gent et al., 2007). Taunton et al. (2003), reported that patellofemoral pain syndrome (PFP) and iliotibial band syndrome (ITBS) are the two most common RRIs; both classified as overuse injuries.

The aetiology of RRIs remains a particularly complex challenge. It has been suggested that a relationship between the loss of muscle strength and mechanical abnormalities linked with RRIs, e.g., increased maximum hip frontal plane angles exists (Noehren et al., 2007). Dierks et al. (2008) and Noehren et al. (2014) both reported a gait signature of increased hip adduction angle in conjunction with decreased hip muscular force production in runners with PFP and ITBS. It remains unknown whether the injuries are the cause or consequence of the mechanical abnormalities and strength deficiency. Changes in mechanics could alter the tissues load absorbing capability or the magnitude of the load experienced, both potentially increasing the cumulative load and likelihood of developing an RRI. Powers (2010) proposed a proximal aetiology model whereby impaired muscular control at the hip, for example increased hip adduction angle, underlie injuries such as PFP and ITBS. A 2 year prospective study by Noehren et al. (2013) found evidence to support the idea of hip aetiology in PFP.

Fatigue has been identified as an extrinsic factor associated with RRI (Rolf, 1995), yet only a handful of studies have examined the effect of acute fatigue on running kinematics in healthy runners. Willson et al. (2015) observed an increase in hip adduction angle in both male and female runners while other studies observed little increase to no change in hip frontal kinematics (Dierks et al., 2010; Bazett-Jones et al., 2013; Brown et al., 2016). These studies however used runs to exhaustion, often performed at the same absolute running speed for all participants. The use of absolute intensities can result in runners performing in different physiological domains resulting in different mechanisms of fatigue (Burnley and Jones, 2018). Moreover, runners seldom perform continuous runs to exhaustion during training, although they do undertake fatiguing high intensity interval training (HIIT) (Laursen, 2010). These HIIT sessions are more effective than medium intensity continuous run (MICR) at improving critical aspects of running performance, i.e., $\dot{V}$O_2_ max (Bacon et al., 2013). Billat et al. (1999) found that frequent use of HIIT sessions (three or more times per week) led to symptoms of overtraining. Despite their undoubted importance, endurance athletes typically use HIIT sessions judiciously, only accounting for 10–15% of weekly training volume (Laursen, 2010). The remaining training being performed at lower intensities to facilitate recovery, often performed continuously, e.g., MICR (Laursen, 2010). It is not known whether HIIT sessions invoke greater changes in gait than continuous running and therefore potentially pose a greater risk of developing RRIs.

To better understand the complexity of gait biomechanics that contribute to the development of overuse RRIs, coordination variability has been employed. Applications of dynamical systems theory have gained attention by investigating running variability through the coordination of coupling joints, where the motion of one joint can influence another (DeLeo et al., 2004). Hamill et al. (2012) proposed that deviating from an optimal variability within the coordination of two joints or segments, to be a risk factor for injury. Hamill et al. (2012) also suggested that injured runners present a reduced coordination variability. Similarly, a very high coordinative variability can contribute to the development of overuse injuries (Hamill et al., 2012). Only two studies have examined the effect of fatigue on running variability using dynamical system theory applications finding both increased and decreased (Miller et al., 2008; Brown et al., 2016) variability in the coupling interactions following a run to exhaustion.

It is unknown whether regular, healthy runners change toward the profile of injured runners by end of a typical training session. Given the multifactorial nature of RRI development a broader research approach is required. The purpose of this study was to observe changes in multiple risk factors (i) muscular strength (ii) kinematics and (iii) joint coupling coordination variability at the beginning and end of a HIIT session and continuous run matched for energy expenditure.

## Materials and Methods

### Participants

Following a power analysis based on the kinematic variables in Dierks et al. (2008) (α = 0.5 and β = 0.20; desired effect size of 0.66) and subsequent institutional ethical approval, 20 healthy, experienced running for at least 2 year, club distance runners (*N* = 10 males; *N* = 10 females) were recruited. Table 1 shows participant characteristics, treadmill speeds and run duration. Participants were excluded if they had not competed in an organised race within the past 2 years, were not part of an affiliated running club, or had experienced any type of lower extremity injury that prevented them from running for more than a week in the past 6 months. Further exclusion criteria included any cardiovascular or neurological conditions, or an allergy to adhesive material. Medical history was pre-screened via a self-reported questionnaire; eligible participants provided informed written consent prior to testing sessions.

**TABLE 1:** Descriptive characteristics of participants, training runs, speeds, durations, $\dot{V}\,O_{2}$ max, speed at lactate turnpoint (sLTP), percentage of $\dot{V}\,O_{2}$ max at sLTP (%$\dot{V}\,O_{2}$ at sLTP), represented as mean ± standard deviation.

	**Female**	**Male**
	**(*n* = 10)**	**(*n* = 10)**
Age (years)	42.24.0	43.84
Height (cm)	164.66.0	181.27.9
Mass (kg)	58.56.2	77.36.5
HIIT Speed (m.s^–1^)	3.90.3	4.60.3
HIIT rep duration (min:sec)	03:2413(s)	02:4716(s)
MICR Speed (m.s^–1^)	3.30.2	3.60.4
MICR duration (min:sec)	32:1502:01	25:5303:40
$\dot{V}\,O_{2}$ max (ml.kg^–1^.min^–1^)	53.65.4	60.54.4
sLTP (m.s^–1^)	3.50.1	3.90.1
%$\dot{V}\,O_{2}$ max at LTP	81.45.5	72.78.1

### Protocol

Each participant completed two treadmill runs on different days with each session separated by 48 h that mimicked different, typical, training intensities. One was a HIIT session, the other a continuous run. All sessions were conducted at the same time of day to minimise diurnal variation. Participants were asked to wear the same footwear throughout and follow their habitual dietary regimen, while refraining from high volume or intensity training during the 48 h separation of testing.

### Preliminary Testing

Initial measurements of mass, stature and all kinanthropometric measures were taken according to ISAK guidelines, by an ISAK qualified practitioner. Participants completed an incremental treadmill (ELG2, Woodway, Germany) test to determine maximum steady state and $\dot{V}$O_2_ max. Expired gas analysis was measured by Cortex Metalyser 3B (Leipzig, Germany), calibrated according to manufacturer’s instructions prior to each test. A 5-min warm-up run was completed to familiarise participants with the treadmill and equipment used for expired gas collections.

The sub-maximal test consisted of a series of incremental 4-min stages at 0% gradient, separated by 60-s recovery with the initial speed was set according to each participants’ current performance level, ranging between 7 and 10 km.h^–1^ (Smith and Jones, 2001). Stages increased by 1 km.h^–1^ until lactate turnpoint was exceeded. Lactate threshold was identified as the first rise in blood lactate above baseline while lactate turnpoint was defined as a second steeper, more sustained rise in blood lactate in accordance with Smith and Jones (2001). Between stages a fingertip capillary blood sample was taken for analysis of blood lactate concentration (Biosen C-line, EKF diagnostics, Germany). Following a 15-min recovery, participants completed a $\dot{V}$O_2_ max test with the initial speed set at 4 km.h^–1^ below the speed at lactate turnpoint at a 0% gradient. The treadmill speed was increased by 0.5 km.h^–1^ every 30 s until volitional exhaustion occurred. Breath by breath $\dot{V}$O_2_ data 30-s averaged, with the highest regarded as $\dot{V}$O_2_ max (Billat et al., 2001). Plateau was determined using the method of Midgley et al. (2009); all runners displayed a plateau.

### HIIT and MICR

The duration and speed of each run was individualised based on $\dot{V}$O_2_ max and lactate turnpoint. The HIIT session was a modification of a protocol previously shown to cause fatigue (James and Doust, 2000). It consisted of six 800 m repetitions at 1 km.h^–1^ below the speed at $\dot{V}$O_2_ max (s$\dot{V}$O_2_ max), with a 1:1 work: rest ratio. The recovery was active, with participants walking at 4 km.h^–1^. The MICR was at halfway between the speed of lactate threshold and speed of lactate turnpoint, defined as the second threshold increase in blood lactate, with the duration set to match the energy expenditure of the HIIT session.

Briefly, the $\dot{V}$O_2_ -running speed relationship was established using the expired gas data from the final 60 s of each sub-maximal stage. From this relationship energy expenditure (kJ.min^–1^), for both the speed of the HIIT intervals and recoveries, was calculated using the method of Shaw et al. (2013). Both energy expenditure values were multiplied by the total time spent at each speed and then summed to give the total energy expenditure. The total energy expenditure from the HIIT session was divided by the energy expenditure for the MICR speed to determine the MICR duration.

### Muscular Strength

In order to examine muscle function and fatigue, maximum force generating capability was examined (Gandevia, 2001), this was performed at the hip and knee musculature pre and post each training session. A handheld dynamometer (Lafayette Instruments, Lafayette, IN, United States) was used to measure maximum voluntary isometric contraction at the hip and knee. The handheld dynamometer was secured at the limb of the participant using a Velcro strap and finally secured to the body with a non-elastic strap placed around an examination table to remove tester strength bias. All hip testing positions were in accordance with Bazett-Jones et al. (2013) and included testing for hip abduction, adduction, extension, flexion, internal rotation, and external rotation movements. Knee extension strength test was performed with participants seated with hip and knee at 90°, with the handheld dynamometer placed on the anterior aspect of the shank, proximal to the ankle joint. Knee flexion was performed with the participants laying down prone with the knee flexed at 30 degrees with the handheld dynamometer placed at posterior aspect of the shank, proximal to the ankle joint. Participants were asked to perform one set of three maximal effort trials for each movement in a 5- s ramp protocol, exerting maximum force against dynamometer during the final 3 s. The highest value was recorded. There was 30 s rest between each effort and a 1–2 min of rest between each muscle group (Bazett-Jones et al., 2013). The strength measures were recorded in Newtons and normalised to body weight. Measurements of lateral epicondyle to greater trochanter were used for hip strength measure normalisation, while lateral malleolus to head of fibula was used for knee movement measures.

### Motion Analysis

Running kinematics were captured using a 14-camera three-Dimensional motion analysis system (Vicon Nexus; Vicon Motion Systems, Ltd., Oxford, United Kingdom) sampling at 500 Hz and calibrated before each session. The data were recorded using a Plugin Gate model and followed the procedure of Riazati et al. (2019). Retroreflective markers were placed bilaterally on the anterior superior iliac spine, posterior superior iliac spine, thigh, lateral epicondyles of the femur, lateral shank, lateral malleoli, base of the 2nd metatarsal, and calcaneus. The markers were carefully placed, by the same researcher throughout, on the desired landmarks with double sided tape and the surrounding base was also taped down with double sided tape. Wand markers were used for the thigh and shank in order to obtain rotational movements of the joints. The participants were given compression leggings to wear to help keep the markers in place. The hip markers were taped around the hip using soft adhesive tape to avoid impeding hip movement and to ensure they remained in place throughout the run. Static capture was performed 3 s prior to treadmill running sessions. This was processed using the static plugin gait model and static subject calibration. For HIIT and MICR, kinematics were recorded for 25 s at the end of the first and start of the final minute of each run.

### Data Analysis

All markers were labelled and marker trajectories were filtered using a fourth order low-pass Butterworth filter via dynamic plug-in gait model with 6 Hz cut-off frequency. Gait identification was achieved through visual inspection of foot strike and toe off for 30 consecutive strides.

Maximum angle (max) and range of motion (ROM) of the hip and knee in the sagittal and frontal planes during the stance phase were extracted. Spatiotemporal variables stride frequency (SF), stride length (SL) and contact time (CT) were also exported for analysis. All motion analysis data were processed using custom written script in Matlab (2018a, The Mathworks, Inc., Natick, MA, United States).

### Variability

Variability of interactions between sagittal (flexion/extension) and frontal (abduction/adduction) planes of motion for the hip and knee joint couplings were analysed using continuous relative phase variability (CRPV) and coupling angle variability (CAV) through vector coding. The selection of these two applications of dynamical system theory for variability was based on Miller et al. (2010), where they found that both methods were valid for examination of running variability. All variability analyses were processed using a custom written script in Matlab (2018a, the Mathworks, Inc, Natick, MA, United States).

### Continuous Relative Phase Variability

The CRPV of interactions at the hip and knee joints were examined during treadmill running from 30 consecutive stance phase cycles. To allow for calculations of the phase angle (φ), phase plots were constructed for each joint motion by plotting angular position (horizontal axis), against angular velocity (vertical axis). Normalisation of the phase plots for every trial was required and outlined in Eqs. 1 and 2, where θ represents joint angle and *i* represents each data point within the stride cycle. Variability of CRP was calculated as standard deviation of the calculated CRP.

(1) $$A\,n\,g\,l\,e\,\left(H\,o\,r\,i\,z\,o\,n\,t\,a\,l\,a\,x\,i\,s\right):\theta \,i=\frac{2*\left[\theta \,i-\min\left(\theta \,i\right)\right]}{\max\left(\theta \,i\right)-\min\left(\theta \,i\right)}-1$$

(2) $$A\,n\,g\,u\,l\,a\,r\,v\,e\,l\,o\,c\,i\,t\,y\,\left(v\,e\,r\,t\,i\,c\,a\,l\,a\,x\,i\,s\right):\omega_{i}=\frac{\omega_{i}}{\max\left\{\left|\omega_{i}\right|\right\}}$$

Angles were normalised to a minimum value of −1 and maximum value of 1, with the horizontal axis in the middle of the range. The largest magnitude of angular velocities normalised to 1 within each stride cycle. The normalised phase plots for the stance phase in each cycle defined the phase angle, φ, as the angle between the right horizontal and a line drawn from the origin to a specific data point (Hamill et al., 1999), as outlined in Eq. 3.

(3) $$\varphi =\tan^{-1}\frac{\omega \,\left(t\right)}{\theta \,\left(t\right)}$$

Continuous relative phase was calculated as the difference between the normalised phase angles (Eq. 4) for the lower extremity interactions joint angles. For the stance phase CRP is represented in a range of [180°, −180°].

(4) $$C\,R\,P\,\left(t\right)=\varphi_{p\,r\,o\,x\,i\,m\,a\,l}\,\left(t\right)-\varphi_{d\,i\,s\,t\,a\,l}\,\left(t\right)$$

Continuous relative phase variability was calculated as the standard deviation of the mean CRP.

### Vector Coding

The CAV from vector coding was calculated after normalisation of the same 30 consecutive stance phase cycles used in CRPV. The calculation steps are adapted from Needham et al. (2014). Coupling angle (CA) was calculated for each instant (i) for the normalised data of the stance phase of the gait cycle. Coupling angle, γ_*i*_, was calculated based on consecutive angles of the proximal and distal joints outlined in Eqs. 5–12. To achieve this the coupling angle was placed through a correction process outlined in Eq. 8. Average coupling angle was calculated based on average horizontal and vertical components at each instant through circular statistics (Eqs. 8 and 9) and corrected again (Eq. 10) to provide the desired range (Hamill et al., 2000; Needham et al., 2014). Length of average coupling is represented as ${\bar{\gamma }}_{i}$ and CAV was calculated using Eqs. 11 and 12.

(5) $$\gamma_{i}=\tan^{-1}\left(\frac{\theta_{D\,\left(i+1\right)}-\theta_{D\,i}}{\theta_{P\,\left(i+1\right)}-\theta_{P\,i}}\right)\,\frac{180}{\pi }\;\theta_{P\,\left(i+1\right)}-\theta_{P\,i}>0$$

(6) $$\gamma_{i}=\tan^{-1}\left(\frac{\theta_{D\,\left(i+1\right)}-\theta_{D\,i}}{\theta_{P\,\left(i+1\right)}-\theta_{P\,i}}\right)\,\frac{180}{\pi }+180\;\theta_{P\,\left(i+1\right)}-\theta_{P\,i}>0$$

$$\gamma_{i}$$

(7) $$=\left\{\begin{array}{cccc} \gamma_{i}=90\,\theta_{P\,\left(i+1\right)}-\theta_{P\,i}=0\,a\,n\,d\,\theta_{D\,\left(i+1\right)}-\theta_{D\,i}>0 & & & \\ \gamma_{i}=-90\,\theta_{P\,\left(i+1\right)}-\theta_{P\,i}=0\,a\,n\,d\,\theta_{D\,\left(i+1\right)}-\theta_{D\,i}<0 & & & \\ \gamma_{i}=-180\,\theta_{P\,\left(i+1\right)}-\theta_{P\,i}<0\,a\,n\,d\,\theta_{D\,\left(i+1\right)}-\theta_{D\,i}=0 & & & \\ \gamma_{i}=U\,n\,d\,e\,f\,i\,n\,e\,d\,\theta_{P\,\left(i+1\right)}-\theta_{P\,i}=0\,a\,n\,d\,\theta_{D\,\left(i+1\right)}-\theta_{D\,i}=0 & & & \end{array}\right\}$$

(8) $${\bar{x}}_{i}=\frac{1}{n}\,\sum_{i=1}^{n}\cos\gamma_{i}$$

(9) $${\bar{y}}_{i}=\frac{1}{n}\,\sum_{i=1}^{n}\sin\gamma_{i}$$

(10) $${\bar{y}}_{i}=\left\{ \begin{array}{cc} \tan^{-1}\left(\frac{{\bar{y}}_{i}}{{\bar{x}}_{i}}\right)\cdot \frac{180}{\pi }\,x_{i}>0,y_{i}>0\; & \\ \tan^{-1}\left(\frac{{\bar{y}}_{i}}{{\bar{x}}_{i}}\right)\cdot \frac{180}{\pi }+180\,x_{i}<0\; & \\ \tan^{-1}\left(\frac{{\bar{y}}_{i}}{{\bar{x}}_{i}}\right)\cdot \frac{180}{\pi }+360\,x_{i}>0,y_{i}<0\; & \\ 90\,x_{i}=0,y_{i}>0\; & \\ -90\,x_{i}=0,y_{i}<0\; & \\ U\,n\,d\,e\,f\,i\,n\,e\,d\,x_{i}=0,y_{i}>0\; & \end{array}\right.$$

(11) $${\bar{\gamma }}_{i}=\sqrt{{\bar{x}}_{i}^{2}+{\bar{y}}_{i}^{2}}$$

(12) $$C\,A\,V_{i}=\sqrt{2\cdot \left(1-{\bar{\gamma }}_{i}\right)}\cdot \frac{180}{\pi }$$

### Statistical Analysis

The data were checked for normality using Q–Q plots, all variables were deemed normally distributed. Mean and standard deviation were calculated for all variables. A 2 × 2 ANOVA with repeated measures were used to examine differences with time (start–end) and run-type (HIIT, MICR) for muscular strength, kinematics, and running variability. Data were tested for sphericity using Mauchly’s test statistic; no adjustments were required, *post hoc* test with Tukey adjustment was used in the event of a significant main effect. Effect sizes were calculated according to Cohen (1988) and interpreted as small (≥0.2), moderate (≥0.5), and large (≥ 0.8) (Cohen, 1988). The level of significance was set at *P* ≤ 0.05. All statistical analysis were performed in SPSS v22.0 (SPSS, Inc., Chicago, IL, United States).

Fatigue effects were considered to have occurred when individual runners experienced changes between start and end of each runs, greater than, or equal to, the minimum detectable change (MDC). The MDC were derived from our own reliability data the tables are attached to the Supplementary Files 1–4 (SDC 1-4, SEM, and MDC values for measures of muscular strength, kinematics, gait, and variability).

## Results

### Muscular Strength

Fatigue was evident following both run-types as all measures of muscular force production decreased with time following both run-types (*P* < 0.05, see Table 2). There were no differences between HIIT and MICR as no interaction for time or run-type were observed (*P* > 0.05). Similarly, effect size comparisons was marginally bigger following HIIT, as for HIIT this ranged from *d* = 0.34 to *d* = 0.69, and from *d* = 0.27 to *d* = 0.58 for MICR. The biggest difference in effect size between the two run-types was observed in hip adduction strength, with *d* = 0.51 in the HIIT compared to *d* = 0.27 for MICR.

**TABLE 2:** Hip and knee body mass-normalised (kg.kg^–1^) strength measures (Mean ± SD) pre vs. post and percentage change in high intensity interval training run (HIIT) and medium intensity continuous run (MICR).

		**Mean ± SD**				**Repeated measure ANOVA results (*P*-value)**		
					
	**Run-type**	**Start**	**End**	**%**	**Effect size**	**Time**	**Run-type**	**Time × Type**
Hip abduction	HIIT	0.508 ± 0.08	0.449 ± 0.09	88.4	d = 0.69	<0.001	0.287	0.551
	MICR	0.490 ± 0.09	0.437 ± 0.09	89.2	d = 0.58			
Hip adduction	HIIT	0.344 ± 0.09	0.301 ± 0.08	87.5	d = 0.51	<0.001	0.233	0.150
	MICR	0.318 ± 0.09	0.296 ± 0.07	93.1	d = 0.27			
Hip internal rotation	HIIT	0.199 ± 0.04	0.178 ± 0.03	89.4	d = 0.59	<0.001	0.218	0.927
	MICR	0.192 ± 0.04	0.172 ± 0.03	89.6	d = 0.57			
Hip external rotation	HIIT	0.249 ± 0.06	0.227 ± 0.05	91.2	d = 0.40	0.002	0.094	0.871
	MICR	0.230 ± 0.06	0.210 ± 0.06	91.3	d = 0.33			
Hip flexion	HIIT	0.409 ± 0.09	0.357 ± 0.08	88.5	d = 0.64	<0.001	0.776	0.732
	MICR	0.403 ± 0.08	0.357 ± 0.07	88.6	d = 0.57			
Hip extension	HIIT	0.330 ± 0.09	0.279 ± 0.08	85.9	d = 0.60	<0.001	0.273	0.231
	MICR	0.321 ± 0.11	0.262 ± 0.11	84.5	d = 0.54			
Knee extension	HIIT	0.509 ± 0.13	0.443 ± 0.09	87.0	d = 0.59	<0.001	0.590	0.313
	MICR	0.482 ± 0.13	0.433 ± 0.09	89.8	d = 0.44			
Knee flexion	HIIT	0.258 ± 0.11	0.222 ± 0.10	86.0	d = 0.34	<0.001	0.512	0.654
	MICR	0.240 ± 0.10	0.214 ± 0.09	89.2	d = 0.27			
								

Individual assessment showed that more runners exhibited a drop in muscular strength greater than, or equal to, MDC in the HIIT compared to MICR run for all strength measures except hip flexion and external rotation. Hip abduction strength was reduced in five runners post HIIT and in three post MICR. For hip adduction strength, one runner experienced a reduction beyond MDC from HIIT but no runners post MICR. Four runners experienced reduced HIR strength beyond MDC post HIIT and three runners post MICR. Knee extension strength reduced beyond MDC for six runners post HIIT and two post MICR. A similar trend was seen in knee flexion, as four runners reduced strength above MDC post HIIT and two post MICR. For hip flexion, no runners exceeded MDC post HIIT, however two runners exceeded MDC post MICR. No runners experienced a reduced muscular strength beyond MDC for hip extension after either run.

### Kinematics

Both runs exhibited fatigue induced kinematic changes that are associated with profiles of injured runners (see Table 3) in the hip but no significant changes (*P* > 0.05) at the knee joint. Hip frontal plane maximum and RoM angles increased significantly with time, *P* < 0.001 and *P* = 0.003. *Post hoc* examination for hip frontal RoM revealed that the HIIT induced a greater effect (*d* = 0.69) compared to the MICR (*d* = 0.43; *P* = 0.004), while no effect for maximum angle between runs was observed. For hip sagittal plane, runners RoM angles increased for both HIIT and MICR significantly with time (*P* < 0.001). There was also a significant change for time and run-type (*P* = 0.019), with HIIT inducing a greater effect (*d* = 0.73) compared to the MICR (*d* = 0.32).

**TABLE 3:** Maximum and range of motion (RoM) angle for Hip and Knee sagittal and frontal plane of movements along with spatiotemporal parameters of stride length (SL), stride frequency (SF), and contact time (CT) represented as means ± SD at start and end high intensity interval training run (HIIT) and medium intensity continuous run (MICR).

			**Mean ± SD**			**Repeated measure ANOVA results (*P*-value)**		
					
	**Variables**	**Run-type**	**Start**	**End**	**ES**	**Time**	**Run-type**	**Time × Type**
**Maximum angle (deg)**								
	Knee sagittal	HIIT	44.26.4	46.88.2°	d = 0.14	0.151	0.337	0.148
		MICR	46.07.3	46.47.5°	d = 0.17			
	Knee frontal	HIIT	−0.074.7	−0.456.3°	d = 0.10	0.817	0.933	0.737
		MICR	0.697.1	1.067.7°	d = 0.05			
	Hip sagittal	HIIT	49.27.0	51.57.1°	d = 0.33	0.429	0.071	0.055
		MICR	47.813.4	47.07.0°	d = 0.07			
	Hip frontal	HIIT	11.44.0	12.74.8°	d = 0.35	<0.001	0.176	0.142
		MICR	10.04.7	12.84.5°	d = 0.46			
**RoM angle (deg)**								
	Knee sagittal	HIIT	24.97.8	27.35.5	d = 0.23	0.097	0.275	0.189
		MICR	26.88.6	27.35.3	d = 0.15			
	Knee frontal	HIIT	4.41.9	5.02.1	d = 0.31	0.277	0.452	0.391
		MICR	5.34.0	5.42.9	d = 0.03			
	Hip sagittal	HIIT	50.14.1	53.14.9	d = 0.73	<0.001	<0.001	0.019
		MICR	45.25.2	46.74.4	d = 0.32			
	Hip frontal	HIIT	11.94.5	15.04.5	d = 0.69	0.003	0.573	0.004
		MICR	12.24.1	14.03.4	d = 0.43			
**Spatiotemporal**								
	SF (strides/min)	HIIT	95.16.3	94.35.0	d = 0.14	0.550	<0.001	0.516
		MICR	88.24.9	88.14.7	d = 0.02			
	SL (m)	HIIT	0.850.1	0.860.1	d = 0.10	0.859	<0.001	0.225
		MICR	0.780.1	0.780.1	d = 0.00			
	CT (s)	HIIT	0.200.2	0.220.2	d = 0.10	0.026	<0.001	0.088
		MICR	0.240.3	0.250.3	d = 0.03			

*Sagittal plane kinematics represent motion of flexion and extension as positive value indicate flexion and negative values indicate extension. Frontal plane kinematics represent abduction and adduction angles as positive values indicate adduction and negative value indicate abduction.*

By contrast to the lack of significant differences at the knee, six runners showed increased maximum knee flexion angle beyond the MDC at the end of the HIIT, while only two runners exhibited a similar change after MICR. For knee flexion RoM angle, the number of runners were two and three for the HIIT and MICR respectively. Two runners exceeded MDC for maximum knee angle in the frontal plane after HIIT while no runners exceeded MDC post MICR. For hip sagittal plane maximum angle, MICR induced an increase above MDC in four runners compared to one at end of HIIT. For RoM angle of hip sagittal plane, a similar pattern occurred with more runners (four) experiencing an increase above MDC in MICR compared to HIIT (three). Maximum hip frontal plane angles showed that three runners exhibited a change above MDC for both HIIT and MICR. However, for hip frontal RoM angles, three runners exceeded MDC as result of HIIT, while no runners exceeded MDC post MICR.

### Spatiotemporal

For spatiotemporal parameters, only CT showed a significant increase with time however the magnitude of change was small (HIIT *d* = 0.10; MICR *d* = 0.03). While the results did not show a significant difference between the two run-types, there is a trend toward CT being more affected in the HIIT than MICR. In either run-type, the results did not show an interaction with time for measures of SF or SL, however there was significant difference between run-type, with *post hoc* test showing the change in HIIT (*P* < 0.001).

The MDC assessment revealed that HIIT caused a pre-post change beyond MDC in more runners compared to MICR for all spatiotemporal parameters. For SF, one runner to exceeded MDC after HIIT but no runners after MICR. Five runners exhibited a reduced SL beyond MDC post HIIT compared with two for MICR. Six runners increased CT beyond MDC at the end of HIIT, with one runner experiencing an increase following MICR.

### Variability

Running variability in all joint couplings of hip and knee joints were increased significantly by time when assessed by CRPV (see Table 4). The results also showed significant changes for time and run-types for all measures, with *post hoc* tests showing HIIT having more effect compared to MICR in the increase in variability. For CAV, only the interaction of Hip_abd/add_ – Knee_abd/add_ exhibited no significant increase in variability. The Hip_flex/ext_ – Knee_abd/add_ interaction showed a significant interaction between time and run-type with *post hoc* test showing the MICR (*d* = 2.88) being more effective than the HIIT (*d* = 1.63) in increasing variability of the runners. For CRPV interactions of Hip_flex/ext_- Knee_flex/ext_, Hip_flex/ext_ -Knee_abd/add_, and Hip_abd/addt_ -Knee_flex/ext_, all bar one runner exceeded the MDC at the end of HIIT compared to 10 runners post MICR. In Hip_abd/add_ – Knee_abd/add_, every runner exceeded the MDC at end of HIIT with 18 runners exceeding it following MICR. Thirteen runners exceed the MDC for CAV of Hip_flex/ext_-Knee_flex/ext_, post HIIT but only one runner following MICR. In Hip_abd/add_-Knee_flex/ext_, 19 runners exceed MDC at end of HIIT with 17 at end of MICR. Both HIIT and MICR caused nine runners to exceed MDC at the end for coupling interaction Hip_abd/addt_ -Knee_abd/add_ but no runners for Hip_flex/ext_ -Knee_abd/ad_.

**TABLE 4:** Running variability examined through continuous relative phase (CRP) and coupling angle (CAV) for the interaction between the knee and hip sagittal and frontal plane motions (means ± SD) at start and end of high intensity interval training run (HIIT) and medium intensity continuous run (MICR).

			**Mean ± SD**			**Repeated measure ANOVA results (*P*-value)**		
					
**Application**	**Couplings**	**Run-type**	**Start**	**End**	**ES**	**Time**	**Run-type**	**Time × Type**
CRPV (deg)								
	Hip_flex/ext_ – Knee_flex/ext_	HIIT	15.213.3	78.89.8	d = 5.44	<0.001	0.004	<0.001
		MICR	21.76.7	36.48.3	d = 1.94			
	Hip_flex/ext_ – Knee_abd/add_	HIIT	5.63.7	45.214.9	d = 3.64	<0.001	0.232	0.004
		MICR	26.525.4	35.927.2	d = 0.35			
	Hip_abd/add_ – Knee_flex/ext_	HIIT	13.49.4	77.49.3	d = 6.84	<0.001	0.023	<0.001
		MICR	22.126.0	41.934.4	d = 0.64			
	Hip_abd/add_ – Knee_abd/add_	HIIT	5.43.4	46.414.9	d = 3.79	<0.001	0.649	0.021
		MICR	12.511.3	41.312.1	d = 2.46			
CAV (deg)								
	Hip_flex/ext_ – Knee_flex/ext_	HIIT	66.55.1	79.21.7	d = 3.34	<0.001	<0.001	0.258
		MICR	62.66.4	73.01.8	d = 2.21			
	Hip_flex/ext –_ Knee_abd/add_	HIIT	67.95.0	73.91.4	d = 1.63	<0.001	0.001	0.006
		MICR	62.25.6	73.70.7	d = 2.88			
	Hip_abd/add_ – Knee_flex/ext_	HIIT	73.90.9	74.10.5	d = 0.27	0.077	0.147	0.279
		MICR	73.41.4	75.53.7	d = 0.75			
	Hip_abd/add_ – Knee_abd/add_	HIIT	71.23.3	74.20.7	d = 1.25	<0.001	0.184	0.162
		MICR	69.63.9	70.416.5	d = 0.06			

*Sagittal plane (X) kinematics represent motion of flexion and extension as positive value indicate flexion and negative values indicate extension. Frontal plane kinematics (Y) represent abduction and adduction angles as positive values indicate adduction and negative value indicate abduction.*

## Discussion

The purpose of this study was to investigate if changes in running gait occurred following two different intensity, energy expenditure matched, training runs. Furthermore, to see if any changes caused the gait to move toward the profile of runners with PFP and ITBS suggesting a potentially presented risk in the development of RRI. Following both HIIT and MICR, runners experienced a drop in muscular strength in the hip and knee musculature. A novel finding was that both gait kinematics and variability concurrently showed signs of exercise induced fatigue following HIIT. These changes in biomechanical profile were toward a gait more akin with runners suffering from PFP or ITBS thereby suggesting an presented risk of RRI development.

Potentially, the most noteworthy finding within the loss of muscular strength was the drop for hip abduction strength, with a reduction of 12.0 and 10.6% after HIIT and MICR respectively. While this study cannot identify causality, the loss of muscular strength at the hip could have contributed to gait alterations. Previous studies have associated reduced hip abductor strength with abnormal hip frontal plane kinematics (Noehren et al., 2007; Taylor-haas et al., 2014) with Dierks et al. (2008) finding a similar pattern to us in strength loss pre-post a run to exhaustion. The drop in muscular strength coincided with changes in running gait signature to be more like that of runners with PFP and ITBS. A loss of muscle function could potentially indicate an inability to control gait or absorb impact force, either of which could potentially present increased injury risk. A similar link between muscle function and injury risk was proposed by Bertelsen et al. (2017), their framework proposed that a reduction in structure specific load tolerance could lead to the development of RRI. This observation was more visible in the HIIT where greater changes in mechanics and gait variability were observed. Interestingly both runs were matched for energy expenditure yet produced a similar drop in strength.

To avoid the problems of the self-selected running speeds used in many previous studies (Dierks et al., 2008, 2010; Bazett-Jones et al., 2013; Brown et al., 2016), we prescribed running speeds based on each individual’s physiological capability. Using a self-selected, but similar duration and speed as our MICR but to exhaustion, Willson et al. (2015) found a corresponding increase in hip adduction RoM angle. Apart from Willson et al.’s (2015) study, our results differ from previous studies on hip kinematics as no differences in peak hip adduction angles have been observed at exhaustion (Dierks et al., 2010; Bazett-Jones et al., 2013; Brown et al., 2016). The runners in Dierks et al. (2008) performed their runs at lower speeds than ours, albeit self-selected, finding no difference in peak hip adduction angle at the end. Dierks et al. (2010) observed minimal changes in RoM and maximum angles of hip adduction. Dierks et al. (2008) and Bazett-Jones et al. (2013) both examined hip strength alongside kinematics finding close to a 6% percent drop in hip abduction and 7% in hip external rotation following a run to exhaustion. Both of those studies only used MICR, to the best of our knowledge this is the first study to have included a HIIT condition.

Unlike many previous studies, we assessed quadriceps strength in conjunction with gait analysis, finding a loss of strength in both knee extensors and flexors at the end of each run. Runner’s knee kinematics were less affected than the hip, with the knee frontal plane RoM angles in our runners similar to those of Dierks et al. (2008, 2010) but higher than Bazett-Jones et al.’s (2013) healthy runners. While our findings were non-significant, the increase in maximum knee frontal angle after HIIT was of greater magnitude compared to MICR. These results could suggest that some runners may have been in dynamic knee valgus by the end of HIIT. Dynamic knee valgus is identified by a combination of increased hip adduction and knee abduction, however it can also be identified solely from increased hip adduction (Powers, 2010). The loss of strength at both the knee and hip could have acted independently or synergistically to affect our runner’s gait. With the increased hip adduction angle, the structures surrounding the knee would likely be under pressure, increasing patellofemoral joint stress, similar to the profile of runners with PFP (Powers et al., 2017).

Alterations to spatiotemporal parameters can also serve as a compensation strategy to accommodate for a reduced ability to tolerate load and produce force rapidly (Nicol and Komi, 2010). In this study, contact time was significantly increased in both run-types. Increased CT can be an indicator of fatiguing processes as greater work is required at the push-off phase (Nicol and Komi, 2010). The gait changes in this study concur with Nicol et al. (1991) hypothesis that a loss of stretch shortening cycle function causes changes in knee flexion, stride length (SL) and ground contact time (CT). The inability of the runners to maintain short contact times can also suggest that the progression of fatigue has impaired the ability to maintain performance (Hayes and Caplan, 2014).

By the end of both HIIT and MICR, the runners had an increased demand for patterns of coordination between the joints and the associated movements. Miller et al. (2008) examined CRPV finding non-significant increases and decreases in variability from start to end of the run. They suggested that the alterations in variability can be attributed to hip muscle dysfunction, although they failed to elaborate on this. The drop of hip and knee muscular strength in our study endorses this, as runners failed to remain stable at the end of both runs. *Post hoc* tests revealed the increased variability was greater post HIIT compared to MICR, with CRPV showing an interaction for run-type in all variables compared to one variable with CAV. As both runs were matched for energy expenditure, and exhibited similar drops in muscular strength, it is likely that both metabolic and non-metabolic mechanisms were in operation. In line with Hamill et al. (2012) we suggest that as runners fatigued, the increased variability reflects a loss of gait control. Future studies are required that focus on the effect of fatigue on gait variability, neuromuscular function and motor control.

### Individual Assessment

The use of *P*-values has been heavily criticised, in particular their misuse for representing definite findings, with some journals no longer allowing their use (Trafimow and Marks, 2015; Greenland et al., 2016). Statistical significance considers whether the probability that a mean response has happened by chance or not, while providing no information on the magnitude of response. Runners however develop RRIs on an individual not collective basis, a recent study by Jauhiainen et al. (2020) corroborates our suggestion by recommending presenting individual of gait pattern. While not having a set criteria, Nicol et al. (1991) reported that following a marathon, two individuals out of seven showed a different kinematic profile that was contrary to the main group findings. Different analytical approaches can yield differing interpretations of the same data set.

To overcome the limitations of using *P*-values and provide objective criteria, we examined the number of individuals who exceeded the MDC. MDC provides a confidence interval based upon measurement error; those who exceed this confidence interval have a change in their score beyond measurement error, therefore it can be considered as a ‘real’ change. Individual assessment showed that not all runners were affected by the run-types. Those who exceeded the MDC developed a biomechanical profile more in line with injured runners seen in previous studies (Powers, 2010) which could represent a risk of developing an overuse RRI.

Individual assessment indicated that while all runners experienced a reduction in muscular strength, not many dropped by more than the MDC. Using *P*-values identified a decrease that while not due to chance, may not be meaningful to all runners. The traditional statistical approach did not reveal a difference in strength loss for the different run-types, yet when using MDC we were able to discern that more runners had a real change post HIIT compared to MICR, except in hip extension strength (see Table 2). This observation is supported by the larger effect sizes post HIIT compared to MICR.

Minimum detectable change values also revealed that in some variables, for example knee sagittal plane kinematics, runners experienced fatigue effects not revealed through using *P*-values. Several runners exhibited an increase in knee sagittal plane angle above MDC at the end of both run-types, with HIIT affecting more runners than MICR. A similar observation was found for knee sagittal plane RoM, however more runners were affected during MICR than HIIT. The only measures where both *P*-values and MDC analysis were similar was for hip frontal plane maximum angle and RoM, where the HIIT run had a greater effect on the runners.

*P-*values did not yield a significant group effect in SL or SF, whereas the MDC assessment was able to show numerous runners experienced decreased SF and increased SL at the end of both run-types, with the HIIT affecting more runners. The observed changes in SF and SL have been associated with greater hip and knee moment at touchdown (Seay et al., 2011) and decreased leg stiffness (Morin et al., 2007). Similarly, the increase in contact time observed in this study may also suggest reduced muscle stiffness (Hayes and Caplan, 2014). Contact time did reveal a significant group change for time but not run-type, however twice as many runners exceeded MDC at end of HIIT compared to MICR. This observation may suggest that the HIIT induced a change in stiffness, providing a possible explanation on why runner’s mechanics and gait variability were affected more from the HIIT.

As the two run-types in this study were matched for energy expenditure, the suggestion based on individual assessment that more runners experienced fatigue inducing changes matching profile of injured runners during the HIIT compared to MICR requires further scrutiny. Further analysis of our data revealed an estimated average stride count during the HIIT was 1782 compared to 2279 strides in MICR. Although not directly measured, this suggests a greater loading rate per ground contact. An increased during HIIT concurs with previous work looking at loading rate (Schache et al., 2011; Dorn et al., 2012) causing a greater loading rate on the musculature and hip and knee joints potentially accelerating fatigue. This is further supported by Petersen et al. (2015) who found lower speeds decreased cumulative load (Petersen et al., 2015). Further work using an instrumented treadmill is required to examine the notion of differentiated accumulated load despite matching for energy expenditure.

Similarly, the fatigue experienced during the HIIT run induced increased gait variability in more runners compared to MICR. Continuous relative phase variability revealed both a statistically significant difference and a greater number of runners beyond MDC for HIIT compared to MICR. For CAV, the only coupling that failed to see any runner change by less than MDC was (Hip_flex/ext_ – Knee_abd/add_), while in all other couplings the number of runners beyond MDC was greater after HIIT than MICR. In line with our hypothesis, as runners fatigued they were unable to maintain their co-ordination which could be seen as a potential precursor in overuse injury (Powers, 2010).

## Conclusion

The main finding of this study was that runners exhibited fatigue induced changes in gait profile at the end of a typical training session. These changes are consistent with those seen in runners with PFP and ITBS and could represent a potential risk of developing a running related overuse injury. This was more prominent following HIIT than moderate intensity continuous running despite an equal energy expenditure. Finally, different statistical approaches provided slightly different findings, based on the observation that injury risk was predominantly individual, we would recommend the use of MDC alongside statistical significance testing for a more sensitive analysis.

## Data Availability Statement

All datasets generated for this study are included in the article/Supplementary  Material.

## Ethics Statement

The studies involving human participants were reviewed and approved by Northumbria University. The patients/participants provided their written informed consent to participate in this study.

## Author Contributions

SR, PH, and NC contributed to the concept/design of the work along with the interpretation of the data. MM contributed to the data analysis. All authors drafted the intellectual content and revised the final version.

## Conflict of Interest

The authors declare that the research was conducted in the absence of any commercial or financial relationships that could be construed as a potential conflict of interest.

## References

[B1] Bacon A. P. Carter R. E. Ogle E. A. Joyner M. J. (2013). VO2max trainability and high intensity interval training in humans: a meta-analysis. *PLoS One* 8:e073182. 10.1371/journal.pone.0073182 24066036 PMC3774727

[B2] Bazett-Jones D. M. Cobb S. C. Huddleston W. E. O’Connor K. M. Armstrong B. S. R. Earl-Boehm J. E. (2013). Effect of patellofemoral pain on strength and mechanics after an exhaustive run. *Med. Sci. Sports Exerc.* 45 1331–1339. 10.1249/MSS.0b013e3182880019 23377834

[B3] Bertelsen M. L. Hulme A. Petersen J. Brund R. K. Sørensen H. Finch C. F. (2017). A framework for the etiology of running-related injuries. *Scand. J. Med. Sci. Sport* 27 1170–1180. 10.1111/sms.12883 28329441

[B4] Billat V. L. Flechet B. Petit B. Muriaux G. Koralsztein J. P. (1999). Interval training at V̇O(2max): effects on aerobic performance and overtraining markers. *Med. Sci. Sports Exerc.* 31 156–163. 10.1097/00005768-199901000-00024 9927024

[B5] Billat V. L. Slawinksi J. Bocquet V. Chassaing P. Demarle A. Koralsztein J. P. (2001). Very short (15s - 15s) interval-training around the critical velocity allows middle-aged runners to maintain V̇O2 max for 14 minutes. *Int. J. Sports Med.* 22 201–208. 10.1055/s-2001-16389 11354523

[B6] Brown A. M. Zifchock R. A. Hillstrom H. J. Song J. Tucker C. A. (2016). The effects of fatigue on lower extremity kinematics, kinetics and joint coupling in symptomatic female runners with iliotibial band syndrome. *Clin. Biomech.* 39 84–90. 10.1016/j.clinbiomech.2016.09.012 27718393

[B7] Buist I. Bredeweg S. W. Bessem B. van Mechelen W. Lemmink K. A. P. M. Diercks R. L. (2010). Incidence and risk factors of running-related injuries during preparation for a 4-mile recreational running event. *Br. J. Sports Med.* 44 598–604. 10.1136/bjsm.2007.044677 18487252

[B8] Burnley M. Jones A. M. (2018). Power–duration relationship: physiology, fatigue, and the limits of human performance. *Eur. J. Sport Sci.* 18 1–12. 10.1080/17461391.2016.1249524 27806677

[B9] Cohen J. (1988). *Statistical Power Analysis For The Behavioral Sciences By Jacob Cohen.* Abingdon: Routledge.

[B10] DeLeo A. T. Dierks T. A. Ferber R. Davis I. S. (2004). Lower extremity joint coupling during running: a current update. *Clin. Biomech.* 19 983–991. 10.1016/j.clinbiomech.2004.07.005 15531047

[B11] Dierks T. A. Davis I. S. Hamill J. (2010). The effects of running in an exerted state on lower extremity kinematics and joint timing. *J. Biomech.* 43 2993–2998. 10.1016/j.jbiomech.2010.07.001 20663506

[B12] Dierks T. A. Manal K. T. Hamill J. Davis I. S. (2008). Proximal and distal influences on hip and knee kinematics in runners with patellofemoral pain during a prolonged run. *J. Orthop. Sport. Phys. Ther.* 38 448–456. 10.2519/jospt.2008.2490 18678957

[B13] Dorn T. W. Schache A. G. Pandy M. G. (2012). Muscular strategy shift in human running: dependence of running speed on hip and ankle muscle performance. *J. Exp. Biol.* 215 1944–1956. 10.1242/jeb.064527 22573774

[B14] Gandevia S. C. (2001). Spinal and supraspinal factors in human muscle fatigue. *Physiol. Rev.* 81 1725–1789. 10.1152/physrev.2001.81.4.1725 11581501

[B15] Greenland S. Senn S. J. Rothman K. J. Carlin J. B. Poole C. Goodman S. N. (2016). Statistical tests. P values, confidence intervals, and power: a guide to misinterpretations. *Eur. J. Epidemiol.* 31 337–350. 10.1007/s10654-016-0149-3 27209009 PMC4877414

[B16] Hamill J. Emmerik R. E. A. Van Heiderscheit B. C. Li L. (1999). A dynamical systems approach to lower extremity running injuries. *Clin. Biomech.* 14 297–308. 10.1016/s0268-0033(98)90092-4 10521606

[B17] Hamill J. Haddad J. M. McDermott W. J. (2000). Issues in quantifying variability from a dynamical systems perspective. *J. Appl. Biomech.* 16 407–418. 10.1123/jab.16.4.407

[B18] Hamill J. Palmer C. Van Emmerik R. E. A. (2012). Coordinative variability and overuse injury. *Sport. Med. Arthrosc. Rehabil. Ther. Technol.* 4:45. 10.1186/1758-2555-4-45 23186012 PMC3536567

[B19] Hayes P. R. Caplan N. (2014). Leg stiffness decreases during a run to exhaustion at the speed at [Formula: see text] O2max. *Eur. J. Sport Sci.* 14 556–562. 10.1080/17461391.2013.876102 24410623

[B20] Hespanhol Junior L. C. Mechelen W. Postuma E. Verhagen E. (2016). Health and economic burden of running-related injuries in runners training for an event: a prospective cohort study. *Scand. J. Med. Sci. Sports* 26 1091–1099. 10.1111/sms.12541 26282068

[B21] Hulteen R. M. Smith J. J. Morgan P. J. Barnett L. M. Hallal P. C. Colyvas K. (2017). Global participation in sport and leisure-time physical activities: a systematic review and meta-analysis. *Prev. Med.* 95 14–25. 10.1016/j.ypmed.2016.11.027 27939265

[B22] James D. V. B. Doust J. H. (2000). Time to exhaustion during severe intensity running: response following a single bout of interval training. *Eur. J. Appl. Physiol.* 81 337–345. 10.1007/s004210050052 10664094

[B23] Jauhiainen S. Pohl A. J. Äyrämö S. Kauppi J. P. Ferber R. (2020). A hierarchical cluster analysis to determine whether injured runners exhibit similar kinematic gait patterns. *Scand. J. Med. Sci. Sport* 30 732–740. 10.1111/sms.13624 31900980

[B24] Laursen P. B. (2010). Training for intense exercise performance: high-intensity or high-volume training? *Scand. J. Med. Sci. Sport* 20 1–10. 10.1111/j.1600-0838.2010.01184.x 20840557

[B25] Linton L. Valentin S. (2018). Running with injury: a study of UK novice and recreational runners and factors associated with running related injury. *J. Sci. Med. Sport* 21 1221–1225. 10.1016/j.jsams.2018.05.021 29853263

[B26] Midgley A. W. Carroll S. Marchant D. Mcnaughton L. R. Siegler J. (2009). Evaluation of true maximal oxygen uptake based on a novel set of standardized criteria. *Appl. Physiol. Nutr. Metab.* 34 115–123. 10.1139/H08-146 19370041

[B27] Miller R. H. Chang R. Baird J. L. Van Emmerik R. E. A. Hamill J. (2010). Variability in kinematic coupling assessed by vector coding and continuous relative phase. *J. Biomech.* 43 2554–2560. 10.1016/j.jbiomech.2010.05.014 20541759

[B28] Miller R. H. Meardon S. A. Derrick T. R. Gillette J. C. (2008). Continuous relative phase variability during an exhaustive run in runners with a history of iliotibial band syndrome. *J. Appl. Biomech.* 24 262–270. 10.1123/jab.24.3.262 18843156

[B29] Morin J. B. Samozino P. Zameziati K. Belli A. (2007). Effects of altered stride frequency and contact time on leg-spring behavior in human running. *J. Biomech.* 40 3341–3348. 10.1016/j.jbiomech.2007.05.001 17602692

[B30] Needham R. Naemi R. Chockalingam N. (2014). Quantifying lumbar – pelvis coordination during gait using a modi fi ed vector coding technique. *Journal of Biomechanics* 47 1020–1026. 10.1016/j.jbiomech.2013.12.032 24485511

[B31] Nicol C. Komi P. V. (2010). “Stretch-shortening cycle fatigue,” in *Neuromuscular Aspects of Sport Performance*, ed. Komi P. V. (Hoboken, NJ: Wiley-Blackwell), 183–215. 10.1002/9781444324822.ch12

[B32] Nicol C. Komi P. V. Marconnet P. (1991). Effects of marathon fatigue on running kinematics and economy. *Scand. J. Med. Sci. Sports* 1 195–204. 10.1111/j.1600-0838.1991.tb00296.x

[B33] Noehren B. Abraham A. Curry M. Johnson D. Ireland M. L. (2014). Evaluation of proximal joint kinematics and muscle strength following acl reconstruction surgery in female athletes. *J. Orthop. Res.* 32 1305–1310. 10.1002/jor.22678 25044305 PMC4314615

[B34] Noehren B. Davis I. Hamill J. (2007). ASB clinical biomechanics award winner 2006 prospective study of the biomechanical factors associated with iliotibial band syndrome. *Clin. Biomech.* 22 951–956. 10.1016/j.clinbiomech.2007.07.001 17728030

[B35] Noehren B. Hamill J. Davis I. (2013). Prospective evidence for a hip etiology in patellofemoral pain. *Med. Sci. Sports Exerc.* 45 1120–1124. 10.1249/MSS.0b013e31828249d2 23274607

[B36] Petersen J. Sorensen H. Nielsen R. O. (2015). Cumulative loads increase at the knee joint with slow-speed running compared to faster running: a biomechanical study. *J. Orthop. Sport. Phys. Ther.* 45 316–322. 10.2519/jospt.2015.5469 25552288

[B37] Powers C. M. (2010). The influence of abnormal hip mechanics on knee injury: a biomechanical perspective. *J. Orthop. Sports Phys. Ther.* 40 42–51. 10.2519/jospt.2010.3337 20118526

[B38] Powers C. M. Witvrouw E. Davis I. S. Crossley K. M. (2017). Evidence-based framework for a pathomechanical model of patellofemoral pain: 2017 patellofemoral pain consensus statement from the 4th international patellofemoral pain research retreat. manchester, UK: part 3. *Br. J. Sports Med.* 51 1713–1723. 10.1136/bjsports-2017-098717 29109118

[B39] Riazati S. Caplan N. Hayes P. R. (2019). The number of strides required for treadmill running gait analysis is unaffected by either speed or run duration. *J. Biomech.* 97:109366 10.1016/j.jbiomech.2019.109366 31604569

[B40] Rolf C. (1995). Overuse injuries of the lower extremity in runners. *Scand. J. Med. Sci. Sports* 5 181–190. 10.1111/j.1600-0838.1995.tb00034.x 7552763

[B41] Schache A. G. Blanch P. D. Dorn T. W. Brown N. A. T. Rosemond D. Pandy M. G. (2011). Effect of running speed on lower limb joint kinetics. *Med. Sci. Sports Exerc.* 43 1260–1271. 10.1249/MSS.0b013e3182084929 21131859

[B42] Seay J. F. Van Emmerik R. E. Hamill J. (2011). Low back pain status affects pelvis-trunk coordination and variability during walking and running. *Clin. Biomech.* 26 572–578. 10.1016/j.clinbiomech.2010.11.012 21536356

[B43] Shaw A. J. Ingham S. A. Fudge B. W. Folland J. P. (2013). The reliability of running economy expressed as oxygen cost and energy cost in trained distance runners. *Appl. Physiol. Nutr. Metab.* 38 1268–1272. 10.1139/apnm-2013-0055 24195628

[B44] Smith C. G. M. Jones A. M. (2001). The relationship between critical velocity, maximal lactate steady-state velocity and lactate turnpoint velocity in runners. *Eur. J. Appl. Physiol.* 85 19–26. 10.1007/s004210100384 11513315

[B45] Taunton J. E. Ryan M. B. Clement D. B. McKenzie D. C. Lloyd-Smith D. R. Zumbo B. D. (2003). A prospective study of running injuries: the vancouver sun run “in training” clinics. *Br. J. Sports Med.* 37 239–244. 10.1136/bjsm.37.3.239 12782549 PMC1724633

[B46] Taylor-haas J. A. Lucas K. C. H. Bates N. A. Myer G. D. Cscs D. Ford K. R. (2014). Reduced hip strength is associated with incrased hip motion during running in young adult and adolescent male. *Int. J. Sports Phys. Ther.* 9 456–467. 25133074 PMC4127508

[B47] Trafimow D. Marks M. (2015). Editorial on null hypothesis testing. *Basic Appl. Soc. Psych.* 37 1–2. 10.1080/01973533.2015.1012991

[B48] Van Gent R. N. Siem D. Van Middeloop M. Van Os A. G. Bierma-Zeinstra S. M. A. Koes B. W. (2007). Incidence and determinants of lower extremity running injuries in long distance runners: a systematic review. *Sport Geneeskd* 40 16–29. 10.1136/bjsm.2006.033548 PMC2465455 17473005

[B49] Willson J. D. Loss J. R. Willy R. W. Meardon S. A. (2015). Sex differences in running mechanics and patellofemoral joint kinetics following an exhaustive run. *J. Biomech.* 48 4155–4159. 10.1016/j.jbiomech.2015.10.021 26525514

[B50] Wiltshire G. R. Fullagar S. Stevinson C. (2017). Exploring parkrun as a social context for collective health practices: running with and against the moral imperatives of health responsibilisation. *Sociol. Heal. Illn.* 40 3–17. 10.1111/1467-9566.12622 28990198

