# Supplementary material for: Fatigue Induced Changes in Muscle Strength and Gait Following Two Different Intensity, Energy Expenditure Matched Runs
Source: Front Bioeng Biotechnol. 2020 Apr 22;8:360. doi: 10.3389/fbioe.2020.00360 (PMC7188949; doi:10.3389/fbioe.2020.00360)
Supplement: Supplementary file 1 [file Table_1.pdf]

Supplementary table 1. Standard error of measurement (SEM), Percentage of SEM expressed as the mean (SEM%). and minimum detectable change (MDC) values for strength measures of hip and knee musculature.

Force Production measures	ICC	SEM	SEM %	MDC
Hip Abduction	0.84	0.031	6.2%	0.087
Hip Adduction	0.86	0.031	9.7%	0.086
Hip Internal Rotation	0.81	0.023	9.8%	0.064
Hip External Rotation	0.84	0.014	7.2%	0.086
Hip Flexion	0.81	0.035	8.7%	0.098
Hip Extension	0.77	0.038	11.6%	0.106
Knee Extension	0.86	0.043	8.6%	0.118
Knee Flexion	0.88	0.030	11.4%	0.183

All measures are normalized to body weight $kg.kg^{-1}$
