# Supplementary material for: Fatigue Induced Changes in Muscle Strength and Gait Following Two Different Intensity, Energy Expenditure Matched Runs
Source: Front Bioeng Biotechnol. 2020 Apr 22;8:360. doi: 10.3389/fbioe.2020.00360 (PMC7188949; doi:10.3389/fbioe.2020.00360)
Supplement: Supplementary file 2 [file Table_2.pdf]

Supplementary Table 2. Comparison Standard Error of Measurement (SEM), Minimum Detectable Changes (MDC), for maximum angles (Max) and range of motion (RoM) of stance phase in sagittal and frontal planes of motion for Knee, and Hip in both run-types.

| | *Sagittal* | SEM | MDC | *Frontal* | SEM | MDC |
|---|---|---|---|---|---|---|
| Knee $_{Max}$ | HIIT | 2.0 | 5.5 | HIIT | 3.0 | 8.3 |
| | MICR | 2.0 | 5.5 | MICR | 2.7 | 7.5 |
| Knee $_{RoM}$ | HIIT | 1.7 | 4.7 | HIIT | 1.6 | 4.4 |
| | MICR | 1.5 | 4.2 | MICR | 2.4 | 6.7 |
| Hip $_{Max}$ | HIIT | 2.9 | 8.0 | HIIT | 1.8 | 5.0 |
| | MICR | 2.9 | 8.0 | MICR | 1.0 | 2.8 |
| Hip $_{RoM}$ | HIIT | 1.9 | 5.3 | HIIT | 1.2 | 3.3 |
| | MICR | 1.4 | 3.9 | MICR | 2.0 | 5.5 |
