# Supplementary material for: Fatigue Induced Changes in Muscle Strength and Gait Following Two Different Intensity, Energy Expenditure Matched Runs
Source: Front Bioeng Biotechnol. 2020 Apr 22;8:360. doi: 10.3389/fbioe.2020.00360 (PMC7188949; doi:10.3389/fbioe.2020.00360)
Supplement: Supplementary file 3 [file Table_3.pdf]

Supplementary Table 3. Comparison Standard Error of Measurement (SEM), Minimum Detectable Changes (MDC) for spatiotemporal parameters of stride frequency (SF), stride length (SL) and contact time (CT) in both run-types.

		SEM	MDC
SF	HIIT	2.838	7.866
	MICR	2.271	6.296
SL	HIIT	0.027	0.074
	MICR	0.026	0.073
CT	HIIT	0.008	0.023
	MICR	0.009	0.025
