# Supplementary material for: Fatigue Induced Changes in Muscle Strength and Gait Following Two Different Intensity, Energy Expenditure Matched Runs
Source: Front Bioeng Biotechnol. 2020 Apr 22;8:360. doi: 10.3389/fbioe.2020.00360 (PMC7188949; doi:10.3389/fbioe.2020.00360)
Supplement: Supplementary file 4 [file Table_4.pdf]

Supplementary Table 4. Comparison of Intraclass Correlation Coefficients (ICC), Standard Error of Measurement (SEM), Minimum Detectable Changes (MDC), for Continuous Relative Phase Variability (CRPV) and Coupling Angle Variability (CAV) of high intensity interval training (HIIT) and medium intensity continuous run (MICR).

		CRPV			CAV		
		ICC	SEM	MDC	ICC	SEM	MDC
$Hip_{flex/ext}$- $Knee_{flex/ext}$	*HIIT*	0.77	2.5	6.9	0.79	2.8	7.8
	MICR	0.84	3.8	10.5	0.77	2.1	5.8
$Hip_{flex/ext}$ - $Knee_{abd/add}$	*HIIT*	0.88	2.6	7.2	0.78	1.7	4.7
	MICR	0.86	4.5	12.5	0.78	1.6	4.4
$Hip_{abd/add}$ - $Knee_{flex/ext}$	*HIIT*	0.89	8.4	23.3	0.84	0.9	2.5
	MICR	0.83	7.4	20.5	0.75	0.7	1.9
$Hip_{abd/add}$ - $Knee_{abd/add}$	*HIIT*	0.92	3.2	8.9	0.76	1.8	5.0
	MICR	0.86	3.7	10.3	0.83	1.2	3.3
